# Salvage Bacteriophage Therapy for a Chronic MRSA Prosthetic Joint Infection

**DOI:** 10.3390/antibiotics9050241

**Published:** 2020-05-09

**Authors:** James B. Doub, Vincent Y. Ng, Aaron J. Johnson, Magdalena Slomka, Joseph Fackler, Bri’Anna Horne, Michael J. Brownstein, Matthew Henry, Francisco Malagon, Biswajit Biswas

**Affiliations:** 1 Division of Infectious Diseases, University of Maryland School of Medicine, Baltimore, MD 21201, USA; Mslomka@som.umaryland.edu; 2 Department of Orthopedic surgery, University of Maryland School of Medicine, Baltimore, MD 21201, USA; Vng@som.umaryland.edu (V.Y.N.); Ajohnson1@som.umaryland.edu (A.J.J.); 3 Adaptive Phage Therapeutics, Gaithersburg, MD 20878, USA; jfackler@aphage.com (J.F.); bhorne@aphage.com (B.H.); Mjbrownstein@gmail.com (M.J.B.); 4 Biological Defense Research Directorate, Naval Medical Research Center, Fort Detrick, MD 21702, USA; Matthew.Henry@mail.mil (M.H.); Francisco.Malagon.civ@mail.mil (F.M.); biswajit.biswas.civ@mail.mil (B.B.)

**Keywords:** bacteriophage therapy, prosthetic joint infection, methicillin resistance *Staphylococcus aureus*, medication induced hepatitis, biofilm

## Abstract

This is a case of a 72 year old male with a chronic methicillin-resistant *Staphylococcus aureus* prosthetic joint infection. After the third intravenous dose of bacteriophage therapy, an unusual, reversible transaminitis prompted stoppage of bacteriophage therapy. Nevertheless, treatment was successful and the patient’s severe chronic infection was eradicated.

## 1. Introduction

Prosthetic joint infections (PJI) are one of the most serious complications of joint replacement surgery. Conservative estimates are that approximately 1–2% of all prostheses will become infected over the life of the implant [1]. The financial burden of treating these infections is staggering. It is estimated that they will cost the US healthcare system $1.62 billion in 2020 [1]. In addition, patients have significant morbidity and mortality as a direct result of our current medical and surgical management to treat these infections [2]. In one study, the five year mortality for prosthetic joint infections is over 20% [2]. PJIs are hard to treat because of bacterial biofilms on the prostheses. Unfortunately, conventional antibiotics have limited ability to cure biofilm infections [3]. This is in part due to the almost dormant metabolic activity of bacteria in biofilms and the architecture of biofilms [3]. The concentrations of conventional antibiotics needed to have activity to bacteria in biofilms can be up to 1000 times higher than the same bacteria in a planktonic state [3]. Therefore, new antimicrobial therapies are needed to treat PJI’s that focus on disrupting biofilms.

In nature, bacteria are prey to viruses called bacteriophages. These viruses have a very narrow spectrum of activity and can be either be lytic or lysogenic. Lytic phages hold the most promise in clinical medicine to treat infections given their ability to cause bacterial lysis. Bacteriophages live in dynamic relationships with bacteria and have coevolved over time. Given this coevolution, bacteriophages have developed innate ability penetrate biofilms and lyse bacteria inside biofilms [4,5,6]. Bacteriophages also have the ability to disrupt the biofilm’s extracellular matrix with the use of depolymerase enzymes [4,5,6]. It is therefore postulated that bacteriophages may be promising agents to help cure clinical biofilm infections. This case report shows the potential benefit of bacteriophage therapy in treating chronic methicillin-resistant Staphylococcus aureus (MRSA) PJIs. It also reinforces the need for phase 1 and 2 clinical trials to further assess the safety and efficacy of bacteriophage therapy in PJIs in combination with standard-of-care antibiotic therapies. 

## 2. Case

**Figure 1:**
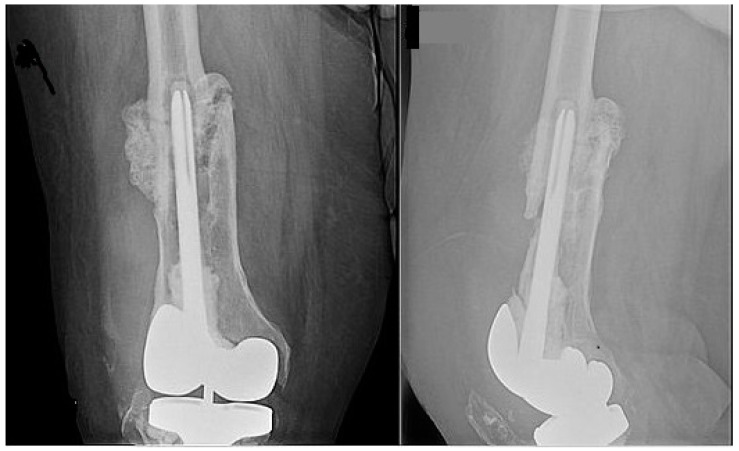
Xray anterior-posterior and lateral views of the right femur showing prior incompletely united distal femoral shaft fracture.

A 72-year-old male with morbid obesity (BMI 41) and hyperlipidemia presented to the University of Maryland Medical Center with a chronic MRSA PJI. Twenty years prior he had a methicillin sensitive *Staphylococcus aureus* (MSSA) septic arthritis and intraosseous abscess of his right knee that was treated with intravenous (IV) cefazolin. One year later, he fractured his distal right femur, requiring open reduction and internal fixation with a lateral plate. He developed severe osteoarthritis over the subsequent decade, and in 2012 he underwent right knee arthroplasty. In 2014, he developed a *Staphlylococcus epidermidis* right knee PJI and underwent debridement, antibiotics, and implant retention (DAIR), but infection recurred, requiring a two-stage revision. In 2016, a fall led to an abrasion over the right knee, and three weeks later, he developed erythema and an effusion. Arthrocentesis revealed MRSA infection. Since the distal femur fracture never fully healed, DAIR was performed. Three weeks later, DAIR was repeated because of worsening symptoms. Hickman catheters were placed and Intraarticular (IA) and IV vancomycin therapy was started. After 5 weeks, vancomycin was changed to IV daptomycin. He completed 3 more weeks of IV daptomycin and a total of 8 weeks of IA vancomycin. His liver function remained normal while on daptomycin. He was then transitioned to chronic oral doxycycline therapy. For two years, oral doxycycline suppressed his infection, but it was stopped and three weeks later the infection recurred. Repeat arthrocentesis culture grew MRSA. Despite resumption of doxycycline, symptoms worsened. Since his distal femur fracture had healed poorly (Figure 1), a standard 2 stage revision was deemed not feasible and the patient refused amputation. He therefore presented to the University of Maryland Medical Center for a second opinion. 

Patient elected to undergo DAIR with IA and IV bacteriophage therapy to salvage the prosthesis. Repeat arthrocentesis grew MRSA. This isolate was sent to Adaptive Phage Therapeutics (APT) and a virulent, lytic bacteriophage was identified that had lytic activity to the isolate. 2.7 × 10^9^ PFU vials of SaGR51φ1 were created by APT. Each vial had less than 1 Endotoxin unit/ml. Please see methods section for more information on isolation and preparation of bacteriophage therapy. Expanded access was granted by the FDA (IND #19274) and by the University of Maryland Baltimore Institutional Review Board (IRB # HP-0087888EU). Consent for emergency use authorization was obtained, and treatment protocol was reviewed by University of Maryland Baltimore Institutional Review Board who deemed this emergency use was acceptable in accordance with 21 CFR 56.102(d) and 21 CFR 56.104(c) [7]. 

**Figure 2:**
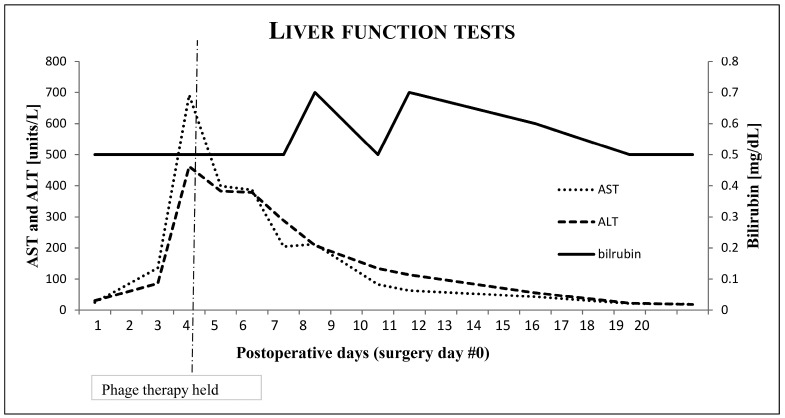
Graph of liver function tests over time of phage therapy.

DAIR was attempted at the University of Maryland Medical Center by an experienced tumor surgeon, Dr. Vincent Y. Ng. Intraoperatively, numerous sinus tracts, gross purulence, and extensive soft tissue infection were present. Extensive erosion of the distal femoral bone stock was present, and gross loosening of the prosthesis was evident. Therefore, prosthesis could not be salvaged. Explant of prosthesis components with placement of static vancomycin and tobramycin spacer was conducted. To allow for femoral reconstruction, clearance of the extensive MRSA infection was required. At the end of surgery, the patient received two doses of IA bacteriophage (5.4 × 10^9^ PFU) in 10 mL of normal saline (NS) and was started on IV daptomycin 1000 mg daily. Daily IV bacteriophage (2.7 × 10^9^ PFU in 50 mL of NS) was started the next day. After the third IV dose of bacteriophage, aspartate aminotransferase (AST) and alanine aminotransferase (ALT) increased to 136 and 86. The next day, AST and ALT were 692 and 462 and bacteriophage therapy was discontinued. Bilirubin, PT/INR, alkaline phosphate, and creatine kinase remained normal. Albumin decreased to 2.7 from baseline of 3.6, as had occurred with his numerous previous surgeries. Daily cumulative doses of Tylenol were less than 1.5 grams. Hepatitis A, B, and C serologies were negative. Tests for influenza, adenovirus, parainfluenza, enterovirus, RSV, EBV, CMV, mycoplasma, chlamydia, and coronavirus were negative. Ultrasound showed hepatomegaly, but no ductal or other abnormalities were seen. Daptomycin was continued at same dose. AST and ALT decreased to normal 10 days later. Figure 2 shows liver function tests over the course of therapy. CRP trended to normal 14 days after surgery. 

IV daptomycin was continued for 6 weeks, and then antibiotics were stopped. Three weeks off antibiotics, the patient underwent a second debridement surgery to confirm clearance of infection and resection of the grossly malunited distal femur fracture and heterotopic bone. Another static vancomycin spacer was inserted. No infection was seen operatively, but another IA bacteriophage dose of 2.7 × 10^9^ PFU in 10 mL of NS was given. Eight cultures from soft tissues, femoral canal, and devitalized bone were all negative. Daily monitoring for 5 days showed no elevations in AST or ALT. Two months later, after optimization of BMI, implantation of a cemented distal femoral megaprosthesis was performed off antibiotics. Given the severity of the previous infection, an additional IA bacteriophage dose of 2.7 × 10^9^ PFU in 10 mL of NS was given. Intraoperative cultures were again negative. No elevations in AST or ALT were observed. One week later, the patient was discharged to receive rehabilitation to strengthen his right lower extremity.

## 3. Methods

### 3.1. Bacterial Isolation

The *Staphylococcus aureus* (SaWF54) bacterial isolate collected from the patient’s synovial fluid was provided to APT to identify a bacteriophage appropriate for therapeutic use. The isolated colony was purified three times on tryptic soy agar media. The colony purified isolate was then screened against *S. aureus* bacteriophage previously collected and stored in APT’s PhageBank™. All media used in the growth of the clinical isolate was certified to be of nonanimal origin.

### 3.2. Bacteriophage Screening Using the Host Range Quick Test (HRQT)

The Host Range Quick Test (HRQT) was used to observe sensitivities against the *S. aureus* PhageBank™ [8]. A liquid culture of the SaWF54 isolate was grown to ~0.1 OD600 and serially diluted to ~1 × 10^7^ CFU/mL in a microtiter plate containing Tryptic soy broth (TSB) and tetrazolium dye. Individual bacteriophage strains were introduced to the patient strain within the wells of the microtiter dish. The interaction of bacteriophage and the patient’s isolate was observed for 48 h. Bacteriophage sensitivity was measured by the inhibition of cellular respiration. Bacteriophage that successfully inhibited or slowed the rate of respiration were also tested for plaquing in a double agar overlay method. The *S. aureus* bacteriophage, SaGR51Φ1, showed inhibition for >30 h relative to the bacterial host control well in the HRQT and was selected for therapy.

### 3.3. Phage Amplification and Purification

The Navy’s medical research center (NMRC) plaque purified the selected bacteriophage three times in preparation for the two-step amplification process [9]. The plaque purified bacteriophage was used to first infect a 100 mL culture of a producer *S. aureus* strain SaGR51 grown to ~0.1 OD600 and infected at a MOI of ~0.1. Bacterial lysis was observed using a spectrophotometer at an O.D600, and the 100 mL lysate was then filtered through 0.22 µm filtration unit.

The small-scale preparation was then used to infect 3.6 L batch of the producer strain SaGR51 under the same growth conditions and MOI as the 100 mL lysate. After the lysis event, the 3.6 L lysate was centrifuged and sequentially filtered.

The bacteriophage was concentrated via PEG8000 10% precipitation overnight at 4 °C and centrifugation at 12,000× *g* for one hour. The pellet was recovered and treated with one volume of chloroform to remove excess cellular debris prior to purification by cesium chloride (CsCl) Continuous Gradient ultracentrifugation. Bacteriophage particles extracted from the CsCl bands were dialyzed four times and 0.22 µm filtered. Approximately, seven (7) mL of this lysate was transferred to APT for further processing [9].

### 3.4. Repurification, Final Therapy Formulation, and Quality Control Testing

After receiving the purified bacteriophage material from NMRC, APT further purified the phage of contaminants such as endotoxins via its proprietary purification methods. After purification, the purified phage was formulated, sterile filtered, and filled in pre-sterilized single-use vials using a fully enclosed, robotically operated ISO 5 classified isolator (VanRx Microcell) in APT’s cleanroom. The lot of final therapeutic vials were quality control tested for sterility (USP <71>), endotoxin levels, and host cell protein levels. The results from initial quality control testing can be found in Table 1.

## 4. Discussion

To this research team’s knowledge, this is the first case of bacteriophage therapy being used successfully as an adjuvant therapy to cure a chronic MRSA PJI. Two other successful bacteriophage treatments of MSSA and Pseudomonas PJIs have been reported [10,11]. Unique to our case is that no chronic suppression antibiotics were used. Little is known about optimal treatment durations or routes of administration to use in PJIs. The plan was to treat longer, but therapy was stopped when significant transaminitis occurred. In spite of this, successful sterilization of the patient’s joint and devitalized bone was achieved with IA and 3 days of IV bacteriophage therapy in combinations with standard of care IV antibiotics for 6 weeks. Given the ability of bacteriophages to self-replicate, perhaps only a few days of bacteriophage therapy are required as an adjunct to surgical debridement. Clinical trials are needed to determine adequate duration of bacteriophage therapy in this setting [10,11]. 

To date, all PJI patients who were successfully treated with bacteriophages required surgical debridement [10,11]. This surgery allows for manual scrubbing of biofilm, ensures that the prosthesis is salvable, and allows instilment of bacteriophages directly to the biofilm. Local dosing of bacteriophages may be vital to clear biofilm infections, but limited data beyond case reports is available [10,11,12]. No adverse events occurred with repeated IA doses, which may be due to limited systemic absorption. Future studies need to be conducted to determine appropriate routes of administration in PJIs. 

The most unique aspect of our case was the transaminitis that occurred following the third IV bacteriophage dose. This seemed to be caused by the bacteriophage therapy. No other liver function derangement was seen, and the transaminitis was reversible and non-life-threatening. Figure 2 shows liver function over the course of bacteriophage therapy. 

The patient had hepatomegaly, but nonalcoholic fatty liver disease could not be proven radiographically, and biopsy was deferred. Over 99% of IV bacteriophage therapy is rapidly cleared by the liver and spleen [13,14,15]. This research team’s theory is that underlying steatosis caused liver macrophages to induce a dysregulated local cytokine response when challenged with large numbers of bacteriophages that needed to be hepatically cleared. This local response could have led to inflammatory changes in the hepatocytes, causing a rise in AST and ALT. This is supported by studies evaluating the role of liver macrophages in steatosis and in older studies evaluating hepatic clearance of bacteriophages [13,14,15]. It is unknown whether mild to moderate elevations in liver enzymes commonly occur following bacteriophage administration. Additionally, it is unknown if continuing IV bacteriophage would have worsened the transaminitis or resulted in adaptation and resolution. For now, intravenous bacteriophage should be used with caution in patients with underlying liver pathology and liver enzymes should be monitored closely. This case is limited because we did not evaluate our patient’s cytokine response to bacteriophage therapy. Future studies should evaluate this response to learn more about the normal human cytokine response to bacteriophage therapy. 

In conclusion, salvage of the patient’s prosthesis was not possible because of severe bone erosion. However, we were able to sterilize the patient’s severe chronic MRSA PJI with a single virulent bacteriophage given IA and IV for 3 days in combination with IV antibiotics. Further PJI studies are needed to establish the optimal duration and route of administration of phages. Bacteriophage therapy has tremendous potential to help cure PJIs, but phase 1 and 2 clinical trials need to be conducted.

## Figures and Tables

**Table 1:** Final therapeutic preparation details provided to this patient for the treatment of *S. aureus* prosthetic joint infections (PJI) infection.

Phage ID	Lot Number	Titer (PFU/mL)	Endotoxin (EU/dose)	Host Cell Protein (ng/mL)	USP <71> Sterility
SaGR51Φ1	eIND19092601	2.7 × 10^9^	<1	<10	PASSED

## References

[1] Kurtz S.M., Lau E., Watson H., Schmier J., Parvizi J. (2012). Economic Burden of Periprosthetic Joint Infection in the United States. J. Arthroplast..

[2] Natsuhara K., Shelton T.J., Meehan J.P., Lum Z.C. (2019). Mortality During Total Hip Periprosthetic Joint Infection. J. Arthroplast..

[3] Mah T.-F.C., O’Toole G.A. (2001). Mechanisms of biofilm resistance to antimicrobial agents. Trends Microbiol..

[4] Leron K. (2015). Targeting Enterococcus faecalis Biofilms with Phage Therapy. Appl. Environ. Microbiol..

[5] Fong S.A., Drilling A., Morales S., Cornet M.E., Woodworth B.A., Fokkens W.J., Psaltis A.J., Vreugde S., Wormald P.J. (2017). Activity of Bacteriophages in Removing Biofilms of Pseudomonas aeruginosa Isolates from Chronic Rhinosinusitis Patients. Front. Microbiol..

[6] Yilmaz C., Colak M., Yilmaz B.C., Ersoz G., Kutateladze M., Gozlugol M. (2013). Bacteriophage Therapy in Implant-Related Infections. J. Bone Jt. Surg. Am. Vol..

[7] Payne K. (2019). Code of Federal Regulations.

[8] Henry M., Biswas B., Vincent L., Mokashi V., Schuch R., A Bishop-Lilly K., Sozhamannan S. (2012). Development of a high throughput assay for indirectly measuring phage growth using the OmniLogTM system. Bacteriophage.

[9] Estrella L.A., Quinones J., Henry M., Hannah R.M., Pope R.K., Hamilton T., Teneza-Mora N., Hall E., Biswajit B. (2016). Characterization of novel Staphylococcus aureus lytic phage and defining their combinatorial virulence using the OmniLog® system. Bacteriophage.

[10] Ferry T., Leboucher G., Fevre C., Herry Y., Conrad A., Josse J., Batailler C., Chidiac C., Medina M., Lustig S. (2018). Salvage Debridement, Antibiotics and Implant Retention (“DAIR”) With Local Injection of a Selected Cocktail of Bacteriophages: Is It an Option for an Elderly Patient With Relapsing Staphylococcus aureus Prosthetic-Joint Infection?. Open Forum Infect. Dis..

[11] Tkhilaishvili T., Winkler T., Müller M., Perka C., Trampuz A. (2019). Bacteriophages as Adjuvant to Antibiotics for the Treatment of Periprosthetic Joint Infection Caused by Multidrug-Resistant Pseudomonas aeruginosa. Antimicrob. Agents Chemother..

[12] Onesa J. (2019). Bacteriophage application for difficult-to-treat musculoskeletal infections: Development of a standard multidisciplinary treatment protocol. Viruses.

[13] Uhr J.W., Weissman G. (1965). Intracellular distribution and degradation of bacteriophage in mammalian tissues. J. Immunol..

[14] Inchley C. (1969). The actvity of mouse Kupffer cells following intravenous injection of T4 bacteriophage. Clin. Exp. Immunol..

[15] Kazankov K., Jørgensen S.M.D., Thomsen K.L., Møller H.J., Vilstrup H., George J., Schuppan D., Grønbæk H. (2018). The role of macrophages in nonalcoholic fatty liver disease and nonalcoholic steatohepatitis. Nat. Rev. Gastroenterol. Hepatol..

